# Determinants of Maternal Satisfaction with Existing Delivery Care at Wolaita Sodo University Teaching and Referral Hospital, Ethiopia

**DOI:** 10.1155/2020/6403123

**Published:** 2020-09-25

**Authors:** Sintayehu Wolka, Sahilu Assegid, Temesgen Tantu, Muluken Gunta, Bereket Duko

**Affiliations:** ^1^Special Support Directorate, Ethiopian Federal Ministry of Health, Ethiopia; ^2^School of Public Health, Institute of Health Sciences Jimma University, Jimma, Ethiopia; ^3^School of Medical Sciences, Wolkite University, Wolkite, Ethiopia; ^4^Wolaita Sodo Health Department, Wolaita Sodo, Ethiopia; ^5^Faculty of Health Sciences, College of Medicine and Health Sciences, Hawassa University, Hawassa, Ethiopia; ^6^School of Public Health, Curtin University, Perth, Western Australia, Australia

## Abstract

**Background:**

Assessing maternal satisfaction on delivery service has significant public health importance to measure the quality of maternal and child care services in a country. Therefore, the objective of this study was to further investigate the determinants of maternal satisfaction on delivery service provided at the Woliata Sodo University Teaching and Referral Hospital, Ethiopia.

**Methods:**

An institutionally based cross-sectional study was employed at the Wolaita Sodo University Hospital, Ethiopia. All mothers who gave birth between March and May 2018 were included in the study. Data were collected through using a pretested and structured interviewer-administered questionnaire. Both bivariate and multivariable logistic regression analyses were performed. A *P* value of <0.05 was used to declare statistical significance.

**Result:**

A total of 398 delivered mothers were included in the study. The rate of maternal satisfaction on existing delivery care was found to be 67.3%. Being less educated (AOR = 5.06, [2.22-11.53]), primigravida (AOR = 3.59, [1.17-11.04]), planned and wanted pregnancy (AOR = 2.74, [1.21-6.18]), having antenatal care follow-up for current pregnancy (AOR = 4.48, [2.04-9.83]), ever used family planning service (AOR = 3.83, [1.95-67.52]), labor duration of less than 6 hours (AOR = 5.96, [2.61-13.57]), and spontaneous vaginal delivery (AOR = 2.82, [1.07-7.42]) were factors significantly associated with maternal satisfaction.

**Conclusion:**

In this study setting, maternal satisfaction was lower compared to other studies. Unreserved effort should be considered for future interventions.

## 1. Introduction

Maternal satisfaction has been commonly defined using the “theoretical models of patient satisfaction” [1]. However, it is a multidimensional concept [2], which can be defined as “positive evaluation of distinct dimensions of health care”, particularly when maternal expectations are met during services provided at labor and delivery wings of health institutions [3].

In the healthcare facilities, women's satisfaction with services provided in the delivery wings can serve as a legitimate indicator to improve maternal and child health care [4]. Therefore, rigorous investigation of maternal satisfaction has become an administrative and practical reality, as it is an important measure of maternal and childcare health services [5, 6].

Clients have explicit desires or requests for the services when they visit healthcare facilities [7, 8]. Therefore, asking them about the care and treatment they received is an important step towards improving the quality of care and for preventing maternal morbidity and mortality [7, 8]. However, many cases of client dissatisfaction can occur due to inadequate discovery of their needs [9].

Nowadays, it is believed that client-centered care would lead to better patient outcomes and provide further information on the provider's success in meeting those client values [6]. Such an assessment of client satisfaction could also add an important “consumer” perspective to the evaluations and feedback of the whole quality improvement agenda within the institution and may serve as one of the quality assessment index [10, 11].

Remarkable progress in improving and expanding primary health care services has been achieved in the past decades [12, 13]. That progress includes providing delivery services free of charge, accessible, and deliverable for all pregnant women [13]. However, providing perceived quality services remains a major challenge. One such challenge is the inadequate exploration of factors that influence maternal satisfaction to delivery service [12, 14].

Client satisfaction surveys represent real-time feedback for providers and show opportunities to improve services and decrease risks [15]. Although some studies have been conducted to assess client satisfaction, limited studies are focused on maternal delivery service satisfaction. For example, an institutionally based cross-sectional study conducted in the Nekemte Specialized Hospital, Ethiopia, reported that the overall satisfaction of mothers with delivery service was 82% [19]. In contrast, 31.3% of mothers were satisfied by the existing labor and delivery care provided at the Gondar University Teaching Hospital, Ethiopia [16]. These findings suggest that numerous determinants may play a significant role in maternal satisfaction in different settings. These also call for the replications of similar studies in different parts of the country to further explore facility-specific determinates of maternal satisfaction in Ethiopia. Therefore, the objective of this study was to further investigate the determinants of maternal satisfaction on the delivery service provided at the Woliata Sodo University Teaching and Referral Hospital, Ethiopia.

## 2. Methods

### 2.1. Study Design and Population

An institution-based cross-sectional study was conducted at the Wolaita Sodo University Referral and Teaching Hospital, Ethiopia. The Wolaita Sodo University Teaching and Referral Hospital is located at Wolaita Sodo Town, which is 327 kilometers far away from Addis Ababa, the capital city of Ethiopia. The hospital reported two thousand and eighty-six delivery cases in the 2016/7 fiscal year. The study was conducted from March 1 to April 25, 2018. All consecutively delivered and discharged mothers at the hospital during the data collection period were included in the study. Mothers who visited the hospital for postnatal service and critically ill were excluded. The sample size of the study was determined by using a single population proportion formula considering the proportion of maternal satisfaction reported in the previous study (*p* = 62%) [17]. Accordingly, a total of 398 mothers were included in this study.

### 2.2. Data Collection

The data collection instrument was drafted using the English language then translated to the Amharic language and back to the English language to check the consistency. Four trained data collectors have collected the data through an exit interview of mothers who were discharged from the postnatal unit. Maternal satisfaction with delivery service was measured using a standard measurement tool constructed by the “World Health Organization (WHO) and the Federal Ministry of Health of Ethiopia” with 36 items [18]. This 5-point Likert scale ranging from “very dissatisfied” to “very satisfied” comprised the following items: health facility or structure-related respondents' satisfaction, process-related respondents' satisfaction, maternal satisfaction on delivery services, the reason for visit, and maternal recommendation on the delivery services of the hospital. To produce a binary outcome, we have merged “very satisfied” and “satisfied” to classify as satisfied, whereas “very dissatisfied”, “dissatisfied”, and “neutral” as unsatisfied. The threshold score for the overall maternal satisfaction was determined by using the demarcation threshold formula, which is {(total highest score-total lowest score)/2} + total lowest score [5]. The total maximum and minimum satisfaction scores were 55 and 15, respectively. The threshold value for satisfaction was found to be 35. Mothers with a total score of ≥35 were categorized as satisfied.

### 2.3. Data Quality Management

The training was given for all data collectors and supervisors on the study instrument, data collection procedure, and also on the objective of the study, individual's right, informed consent, and techniques of the interview. Before starting the actual survey, the questionnaire was pretested on 20 mothers from other hospitals. Cronbach's alpha was computed to assess the internal consistency of the instrument (*α* = 0.88). Regular supervision and frequent communication were done during the data collection.

### 2.4. Data Processing and Analysis

First descriptive statistics were performed then followed by bivariate logistic regression to identify the determinants of maternal satisfaction with delivery service, and variables that had a *p* value <0.05 were considered as significant variables for the multivariate logistic regression analysis. Finally, multivariate logistic regression analysis was applied to describe the relative effect of independent variables on the overall maternal satisfaction. The possibility of multicollinearity and interaction between independent variables was checked. The adjusted odds ratios (ORs) were used to report the association of explanatory variables with the outcome variable at a *p* value of <0.05 and 95% CI.

## 3. Results

### 3.1. Sociodemographic Characteristics of the Study Participants

The mean age of the study participants was 28 ± SD (6.78) years. Among the study participants, the majority of 263 (66.1%) were within the age group of 20-34 years, 282 (45.7%) were housewives, 92(24.9%) were government employees, 309 (77.6%) were married, and 64 (16.1%) were achieved college diploma or more. The mean household income of delivering mothers was 25.39 USD (Table 1).

### 3.2. Obstetric Characteristics of Respondents

Of the respondents, 250 (62.8%) decided the place of delivery by themselves, 258 (64.8%) were given birth through SVD, 333 (83.7%) planned their pregnancy, and 40 (10.1%) were multipara. Mothers who stayed less than 2 years after their last delivery was found to be 72 (30%). Most importantly, 107 (27%) of the study participants gave birth while they were under 18 years of age (Table 2).

### 3.3. Maternal Satisfaction with the Delivery Care Service

**Figure 1:**
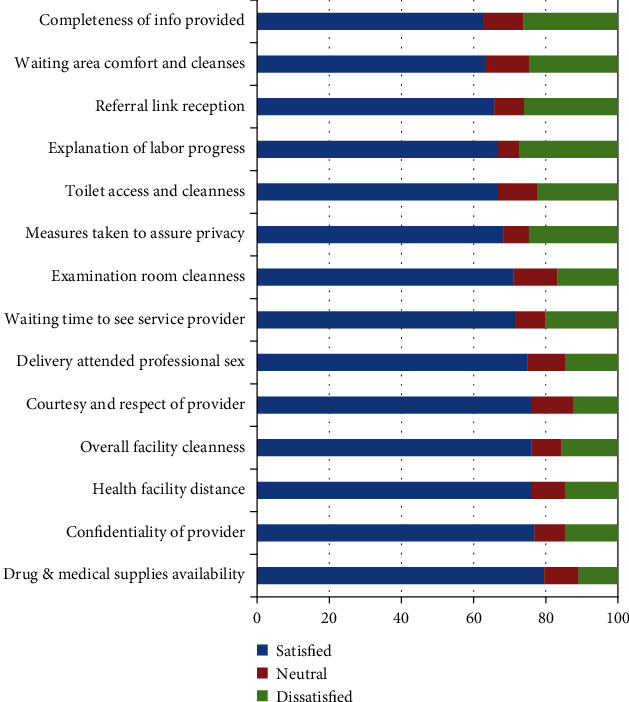
Major dimensions of care and average satisfaction scores by delivering mothers at the Wolaita Sodo University Teaching and Referral Hospital, South Ethiopia, March 2018.

A total of 268 (67.3%) mothers were satisfied with the delivery service. As presented in Figure 1, support is given immediately after delivery (79.6%), availability of required drugs and medical supplies (79.6%), the confidentiality of service provider (76.8%), courtesy and respect of service provider (75.9%), and waiting time to see service provider (71.4%) and overall facility cleanness (76%) which were the major sources of satisfaction, whereas toilet cleanness (22.4%), referral linkage (26%), waiting area comfort cleanness (24.7%), and completeness of the information provided (26.2%) were the major sources of maternal dissatisfaction.

### 3.4. Predicators of Maternal Satisfaction with Delivery Care Service in Multivariate Analysis

Those study participants who completed secondary school from grade (8–12) were 5.06 times more likely to report maternal satisfaction when compared to those who graduated with a diploma and above [AOR = 5.06; 95% CI: (2.22-11.53)]. The odds of reporting maternal satisfaction with delivery services provided increase by 3.09-fold in study participants who gave their first birth [AOR = 3.59; 95% CI: (1.17-11.04)]. Those mothers who reported their positive intention to current birth (wanted pregnancy) were 2.74 times more likely to be satisfied with delivery services when compared to their counterparts [AOR = 2.74; 95% CI: ([1.21-6.18)]. Having regular ANC follow-ups had a positive impact on maternal satisfaction with delivery services. For example, the odds of reporting maternal satisfaction with delivery services increase in women who had ANC follow-up for the current birth [AOR = 4.48; 95% CI: (2.04-9.83)]. Similarly, family planning [AOR = 3.83; 95% CI: (1.95-7.52)], labor duration less than 6 hours [AOR = 5.96; 95% CI: (2.61-13.57)], labor duration of 6-12 hours [AOR = 4.16; 95% CI: (2.04-8.45)], and spontaneous vaginal delivery (SVD) [AOR = 2.82; 95% CI: (1.07-7.42)] were significantly associated with maternal satisfaction (Table 3).

## 4. Discussion

The rate of maternal satisfaction with delivery services reported in our study was in line with the reports from other studies conducted to investigate maternal satisfaction among healthcare institutions in the Amhara (61.9%) and Jimma (65.2%) regions [8, 17] in Ethiopia. However, the finding of the current study was much lower when compared to institutionally based cross-sectional studies conducted in Nekemte Specialized Hospital (82%) [19], in Gamo-Goffa (79.1%) [19], and Debre Markos (81.7%) [20], whereas the finding of this study was much higher than the report from the Gondar University Teaching Hospital, Ethiopia (31.3%) [16]. This difference may be because of a real variation in the quality of services provided, the expectation of mothers, or the type of health facilities and also, the difference might be attributed to the fact that this study was conducted in a referral teaching hospital where sophisticated registration system and high flow of clients are eminent [19]. Moreover, it may be due to variations in the literacy of respondents, cultural diversity and parameter used to measure satisfaction, and the techniques used to compute overall maternal satisfaction.

Privacy is the major requirement for women undergoing delivery because it results in shame and discomfort during a physical examination and the whole delivery procedure. Women in the delivery procedure should receive complete information and enough explanations about the procedure they are undergoing during and after delivery as they have the right to know and ask questions. Also, she will have a chance to be part of the decision process and grant her preference. Finding from this study indicated that participants who felt the inadequacy of privacy and explanation of labor progress during delivery care were dissatisfied with the delivery care service. This finding is inconsistent with a study from India [22].

Providers' respect and courtesy during delivery care have an obvious positive effect on women's satisfaction as it allows easy communication and development of trust in the service received. A review of randomized controlled trials in developing countries found that women who had continuous encouragement and information on labor progress were less likely to be dissatisfied with their delivery process [21]. Respondents of the current study who felt respected and confidential with the competence of service providers were more satisfied than their counterparts. This is also matched to findings from Kenya and Pakistan studies [21, 22].

Facilitated referral linkage between health care facilities is the number one reason that is found to increase client satisfaction [23], especially mothers who are in labor pain should be served promptly as pain arising from the labor is unbearable. The result from this study indicated that respondents who felt there is a gap in accepting referral link immediately as they arrived were less satisfied. This result is consistent with a qualitative study finding from Ghana [24]. In many previous studies, service cost was the main source of client dissatisfaction; studies from different parts of developing countries show the same [17, 19, 22]. The current study revealed only 8.3% of the respondents were asked to pay, indicating the appropriateness of the measure taken by the government to provide exempted service, especially on maternal health care services.

Unlike other studies, the current study did not find any association between participant's age, occupation, and household income with maternal satisfaction, unlike several studies that indicated that younger mothers and those with medium income tend to be more satisfied than the older ones [8, 21].This may be due to differences in sociodemographic and other characteristics between respondents across various settings.

The educational status of delivering mothers was a significant predictor of maternal satisfaction. Less educated mothers tend to have higher satisfaction compared to educated ones. Mothers who attended secondary education were about five times more satisfied than those who hold a diploma and above. This may be due to high expectation forecasted or underestimation of safe delivery by more educated mothers throughout the care process. This finding is consistent with the finding from Ethiopia [23] and studies from Pakistan [17] and western Nepal [25].

Different findings are forwarded regarding the number of child women who have and her satisfaction during delivery service. The current study revealed that primi mothers were more satisfied than multiparous. This may be secondary to the obvious reason that primi mothers are more fearful and curious about the whole delivery procedure and labor pain which in turn may be resulted in happiness-induced satisfaction after delivery, i.e., positive influence following the completion of labor and the joy of childbirth. The finding is also similar to other studies from Ethiopia [23].

Wanted status of pregnancy was also found to be a significant predictor of satisfaction. Mothers who wanted their pregnancy were about three times more satisfied than those who did not. This may be due to perceived confidence in socioeconomic and cultural readiness towards welcoming already the expected baby. On the other hand, having planned pregnancy may be connected with better knowledge and awareness of health services as well as familiarity with health facilities before delivery service [8]. This finding is consistent with findings from Ethiopia [17, 23] and Kenya [21].

ANC service attending mothers have a pretty good chance of having the opportunity to be familiar with the health care facility and probably a chance to know their attendant which will in turn help to build intimacy. The result from this study revealed mothers who had ANC follow-up that had fourfold increased satisfaction compared to those who did not. This is in line with studies conducted in Ethiopia [8, 23]. This implies that access to counseling sessions during prenatal visits about the benefits of institutional delivery may result in shaping a harmonious bond in which providers and mothers become friendly during the delivery care process.

This study indicated that laboring duration was significantly associated with maternal satisfaction. Similarly, a study conducted in other parts of Ethiopia also showed that the satisfaction of mothers who stayed in labor pain for less than 12 hours was around 4 times higher. Similarly, the additional qualitative study also pointed having long labor influences the satisfaction of mothers negatively [20]. This may be due to early relief from pain related to labor. Mothers who gave spontaneous vaginal delivery are with higher odds of satisfaction than those who gave birth by Cesarean section. This finding is in line with a study from Nepal [25]. This may be due to the mother's perception of safe spontaneous vaginal delivery to be an indicator of good and competent care.

An important finding of this study is there was a significant association between ever family planning utilization and maternal satisfaction. The odds of reporting maternal satisfaction increase by fourfold in mothers who used family planning services when compared to their counterparts. This is maybe due to a sense of pride in realizing the benefit of family planning service, thereby enjoying the fruit of already planned pregnancy and delivery or being familiar with the health facility or with service providers. The EDHS report of 2016 presented that family planning use helps women to space the births of their child, which benefits the health of the mother and their child [7].

## 5. Conclusion

The rate of maternal satisfaction with the existing delivery services in the current study setting was relatively lower. Being less educated, primigravida, wanted pregnancy, ANC follow-up for current pregnancy, utilization of family planning service, with shorter duration of labor, and spontaneous vaginal delivery are significantly associated with maternal satisfaction. Further studies supplemented with qualitative design should be conducted to assess maternal satisfaction with the delivery service. Therefore, unreserved effort should be considered by healthcare facilities for intervention on increasing ANC uptake to reach all women, FP service utilization to prevent unintended pregnancy, and promoting active management of labor.

## 6. Limitations of the Study

This study has limitations which is inherent to satisfaction studies. The first is the tendency of respondents to answer positively to questions about satisfaction because of felt favor to the care providers resulting in social desirability bias. Preexisting expectations of mothers and their preferences may have resulted in over- or underexpression of their satisfaction.

## Figures and Tables

**Table 1:** Sociodemographic characteristics of the respondents at the Wolaita Sodo University Teaching and Referral Hospital, South Ethiopia, March 2018 (*n* = 398).

Variables	Category	Frequency	%
Age of respondents	16-20	63	19.6
	21-34	264	66.1
	35-49	71	14.3
Marital status	Single	49	12.4
	Married	309	77.6
	Widowed	9	2.2
	Divorced and separated	31	7.8
Religion	Orthodox	204	51.3
	Protestant	142	35.7
	Muslim	45	11.3
	Others	7	1.8
Educational status	No formal education	83	20.8
	Grades 1-7	97	24.4
	Grades 7-12	154	38.7
	Diploma and above	64	16.1
Occupation	Government employee	99	24.9
	Private employee	54	13.6
	Housewife	182	45.7
	Others	63	15.9
Family monthly income	<500 ETB	92	23.1
	500-1500 ETB	265	66.6
	>1500 ETB	41	10.3
Residence	Urban	232	58.3
	Rural	166	41.7

**Table 2:** Obstetric characteristics of respondents at the Wolaita Sodo University Teaching and Referral Hospital, South Ethiopia, March 2018 (*n* = 398).

Variables	Category	Frequency	(%)
Age at first pregnancy	<18 years	107	26.9
	≥18 years	291	73.1
Parity	One	157	39.4
	Two-four	201	50.1
	Five and above	40	10.1
Reason for current visit	Planned	260	65.3
	Referred	138	34.7
Place of delivery decision	Mother herself	250	62.8
	Husband	90	22.6
	Neighbors and family	58	14.6
Wanted status of pregnancy	Wanted	333	83.7
	Unwanted	65	16.3
Duration of labor	<6 hours	147	36.9
	6-12 hours	157	39.4
	>12 hours	94	23.6
Mode of delivery	SVD	258	64.8
	Assisted instrumental	83	20.9
	Cesarean section	57	14.2
Maternal outcome	Normal	337	84.7
	With complication	61	15.3
Foetal outcome	Normal	370	93
	Died	20	7
Duration of stay after last delivery (*n* = 241)	≥ 3 years	169	70.1
	< 2 years	72	29.9
ANC follow-up for current pregnancy	Yes	321	80.7
	No	77	19.3
Family planning use Hx	Yes	225	56.5
	No	173	43.6
Place of last delivery (*n* = 241)	Health facility	167	69.3
	Home	74	30.7

**Table 3:** Predicators of maternal satisfaction with delivery service among clients at the Wolaita Sodo University Teaching and Referral Hospital, South Ethiopia, March 2018.

Variables	Satisfied	Dissatisfied	COR (95% CI)	AOR (95% CI)	*p* value
Educational status					
No formal education	38 (45.8%)	45 (54.2%)	0.38 [0.19-0.76]^∗^	1.37 [0.58-3.27]	0.472
Grades 1-7	57 (58.8%)	40 (41.2%)	0.64 [0.33-1.26]	1.64 [0.71-3.76]	0.244
Grades 8-12	129 (83.8%)	25 (16.2%)	2.34 [1.19-4.63]^∗^	5.06 [2.22-11.53]^∗∗^	< 0.001
Diploma and above	44 (68.8%)	20 (31.2%)	1	1	
Parity					
One	109 (69.4%)	48 (30.6%)	4.72 [2.24-9.92]^∗^	3.59 [1.17-11.04]^∗∗^	0.026
Two–four	146 (72.6%)	55 (27.4%)	5.51 [2.65-11.45]^∗^	2.39 [0.86-6.63]	0.094
Five and above	13 (32.5%)	27 (67.5%)	1	1	
Pregnancy status					
Wanted	250 (75.1%)	83 (24.9%)	7.28 [4.46-11.80]^∗^	2.74 [1.21-6.18]^∗∗^	0.015
Unwanted	18 (27.7%)	47 (72.3%)	1	1	
ANC follow up for current pregnancy					
Yes	248 (77.3%)	73 (22.7%)	9.68 [8.26-22.69]^∗^	4.48 [2.04-9.83]^∗∗^	< 0.001
No	20 (26%)	57 (74%)	1	1	
Reason for visit					
Planned delivery	193 (74.2%)	67 (25.8%)	2.42 [1.56-3.74]^∗^	1.80 [0.99-3.25]	0.051
Referral for delivery	75 (54.3%)	63 (45.7%)	1	1	
Family planning ever used					
Yes	158 (77.1%)	47 (22.9%)	2.54 [1.65-3.91]^∗^	3.83 [1.95-7.52]^∗∗^	< 0.001
No	110 (57%)	83 (43%)	1	1	
Duration of labor					
< 6 hours	125 (85%)	22 (15%)	13.39 [7.11-25.22]^∗^	5.95 [2.61-13.57]^∗∗^	< 0.001
6-12 hours	115 (73.2%)	42 (26.8%)	6.45 [3.66-11.37]^∗^	4.16 [2.04-8.45]^∗∗^	< 0.001
>12 hours	28 (29.8%)	66 (70.2%)	1	1	
Mode of delivery					
Spontaneous vaginal delivery	193 (74.8%)	65 (25.2%)	3.54 [1.96-6.40]^∗^	2.82 [1.07-7.42]^∗∗^	0.035
Assisted instrumental	49 (59%)	34 (41%)	1.72 [0.87-3.39]	1.31 [0.58-2.93]	0.515
Cesarean section	26 (45.6%)	31 (54.4%)	1	1	

## Data Availability

All the data pertinent to this study are presented in the manuscript. Raw data can be presented by the principal investigator upon reasonable request.

## Abbreviations

CS: Cesarean section
SVD: Spontaneous vaginal delivery.
